# A directed learning strategy integrating multiple omic data improves genomic prediction

**DOI:** 10.1111/pbi.13117

**Published:** 2019-04-14

**Authors:** Xuehai Hu, Weibo Xie, Chengchao Wu, Shizhong Xu

**Affiliations:** ^1^ College of Informatics Agricultural Bioinformatics Key Laboratory of Hubei Province Huazhong Agricultural University Wuhan Hubei China; ^2^ Department of Botany and Plant Sciences University of California Riverside CA USA

**Keywords:** directed learning, genetic features, genomic prediction, LASSO, multiple omic data

## Abstract

Genomic prediction (GP) aims to construct a statistical model for predicting phenotypes using genome‐wide markers and is a promising strategy for accelerating molecular plant breeding. However, current progress of phenotype prediction using genomic data alone has reached a bottleneck, and previous studies on transcriptomic and metabolomic predictions ignored genomic information. Here, we designed a novel strategy of GP called multilayered least absolute shrinkage and selection operator (MLLASSO) by integrating multiple omic data into a single model that iteratively learns three layers of genetic features (GFs) supervised by observed transcriptome and metabolome. Significantly, MLLASSO learns higher order information of gene interactions, which enables us to achieve a significant improvement of predictability of yield in rice from 0.1588 (GP alone) to 0.2451 (MLLASSO). In the prediction of the first two layers, some genes were found to be genetically predictable genes (GPGs) as their expressions were accurately predicted with genetic markers. Interestingly, we made three dramatic discoveries for the GPGs: (i) GPGs are good predictors for highly complex traits like yield; (ii) GPGs are mostly eQTL genes (*cis* or *trans*); and (iii) trait‐related transcriptional factor families are enriched in GPGs. These findings support the notion that learned GFs not only are good predictors for traits but also have specific biological implications regarding regulation of gene expressions. To differentiate the new method from conventional GP models, we called MLLASSO a directed learning strategy supervised by intermediate omic data. This new prediction model appears to be more reliable and more robust than conventional GP models.

## Introduction

A major goal of genetics is to investigate the relationship between genotypes and phenotypes. Although genome‐wide association studies (GWAS) have successfully identified thousands of trait‐associated genetic loci for human diseases and plant agronomic traits in the past decade (Maurano *et al*., 2012), their abilities of predicting phenotypes remain limited because detected significant loci can only explain a small proportion of trait variation (De Los Campos *et al*., 2010). Especially for polygenic traits that are affected by a large number of interacting genes with small effects, very few loci are detectable via GWAS, resulting in failed predictions.

Alternatively, genomic prediction (GP) aims to construct a statistical model for predicting phenotype using genome‐wide markers jointly (Hayes and Goddard, 2001). In plant breeding, it takes advantage of early selection of potential valuable varieties with their genotypes only (de los Campos *et al*., 2013a). Especially, for hybrid breeding that has been proven to be the most efficient way for increasing production in crop breeding, GP has an apparent advantage by predicting and selecting superior hybrid descendants based only on genotypes of their parents (Xu *et al*., 2014). Therefore, successful development of a GP model will greatly accelerate breeding process and save tremendous resources, which makes it a promising strategy for efficient breeding in plants (Desta and Ortiz, 2014).

Published statistical methods for GP can be classified into three categories: parametric methods including genomic best linear unbiased prediction (GBLUP) (de los Campos *et al*., 2013b; VanRaden, 2008; Xu *et al*., 2014) and least absolute shrinkage and selection operator (LASSO) (Li and Sillanpää, 2012; Usai *et al*., 2009), semiparametric methods such as reproducing kernel Hilbert spaces regression (RKHS) that are embedded in linear models (Gianola *et al*., 2006) and statistical learning methods including support vector machine (SVM) (Maenhout *et al*., 2007), neural network (Gianola *et al*., 2011; González‐Camacho *et al*., 2012) and random forest (RF) (Holliday *et al*., 2012). Although many different statistical methods have been tested for GP, there is no consistent conclusion about which method is the best because the predictability of a method heavily depends on traits and populations studied (de los Campos *et al*., 2013a; Riedelsheimer *et al*., 2012; Xu *et al*., 2016). A common problem of the current methods is the inadequate power of capturing higher order information of gene interactions (De Los Campos *et al*., 2010).

In addition to DNA markers, advanced molecular techniques are generating more intermediate phenotypes, such as transcriptomic and metabolomic data, which act as bridges between genotypes and phenotypes and may reflect the information of gene interactions. Some works employed transcriptome to test phenotype predictions recently (Frisch *et al*., 2010; Zenke‐Philippi *et al*., 2016). For example, Frisch *et al*. (2010) attempted to use transcriptome‐based distance (TBD) profiles to predict hybrid performance and heterosis in maize and concluded that transcriptomic prediction (TP) was more precise than GP. In addition to transcriptome, metabolome has also be used for prediction because it is closer to phenotype than transcriptome (Keurentjes, 2009; Riedelsheimer *et al*., 2012; Xu *et al*., 2016). Notably, Riedelsheimer *et al*. (2012) carried out a systematic study using 56 110 SNP markers and 130 metabolites in hybrid maize. The result of GP was slightly better than that of metabolomic prediction (MP), and the reason might be the huge difference in the number of features (56 110 SNPs vs 130 metabolites). More recently, Xu *et al*. (2016) compared predictabilities of GP, TP and MP for six different prediction models and a consistent conclusion was that MP equipped with additive and dominance effects from parental metabolites achieved significant improvements (almost twice for yield) in predicting hybrid rice when compared with GP.

While the current progress of phenotype prediction using genomic data alone has reached a bottleneck, TP and MP are good alternatives suggested by recent studies. However, current TP and MP have ignored genomic information, which is useful and sometimes is a major determinant of phenotype especially for those with high heritabilities. For the sake of maximizing the use of multiple omic data, integrating multiple omic data into one GP model is urgently required. Here, we proposed such an integration strategy, that is multilayered least absolute shrinkage and selection operator (MLLASSO), for improving GP. The key idea of MLLASSO is to implement an innovative directed learning strategy that allows us to learn three layers of genetic features (we denote them as ‘GFs.1L’, ‘GFs.2L’ and ‘GFs.3L’ in the rest of this study) supervised by transcriptomic and metabolomic data using genetic variants as instrumental variables (IV), which has been proven to be an efficient statistical technique to select and estimate optimal instruments (Lin *et al*., 2015). Our approach is still GP because it only requires genomic markers as the input data, but it differs from the traditional GP in that it integrates transcriptomic and metabolic information into a single model and may capture higher order information of gene interactions.

## Results

### Transcriptome or metabolome provides substantial additional information in predicting yield

A rice recombinant inbred line (RIL) population with previously reported data sets was used in this study, which contains 210 lines with 1619 bins (synthetic markers), 24 994 gene expressions (transcripts), 1000 metabolites and four agronomic traits including yield per plant, tiller number per plant, grain number per panicle and 1000 grain weight (KGW). A more complete description of the data is given in the Experimental procedures section.

At first, we used the LASSO method to perform GP with all 1619 bins and found that the predictability of yield was 0.1588, which was evaluated by a 10‐fold cross‐validation (10‐fold CV) test (Table 1). Meanwhile, we performed TP and MP by using 24 994 transcripts and 1000 metabolites independently and the predictabilities of TP and MP were 0.4869 and 0.4593, respectively (Table 1). This implies that transcriptome and metabolome provide substantial additional information in predicting polygenic traits like yield, and it also motivates us to ask how to integrate useful intermediate omic data into an advanced GP model.

**Table 1:** Predictabilities of ten different inputs using 10‐fold cross‐validation test

Input	Dimension	Yield	Tiller	Grain	KGW
LASSO (genome)	1619	0.1588	0.4736	0.3837	0.7625
LASSO (transcriptome)	24 994	0.4869	0.3178	0.5595	0.6903
LASSO (metabolome)	1000	0.4593	0.3800	0.4775	0.5415
LASSO (representative transcriptome)	5467	0.5012	0.3433	0.5080	0.7096
MLLASSO (GFs.1L, *α* = 0.0)	~4950	0.1189	0.4612	0.3684	0.7509
MLLASSO (PT.1L.GPGs, *α* = 0.55)	~1880	0.1900	0.4358	0.2982	0.7231
MLLASSO (PT.2L.GPGs, *β* = 0.25)	~1700	0.2207	0.4541	0.4095	0.7563
MLLASSO (PT.1L+2L.GPGs, *β* = 0.0)	~5130	0.1894	0.4663	0.3985	0.7597
MLLASSO (PT.1L+2L.GPGs, *β* = 0.65)	~1900	0.2451	0.4461	0.3920	0.7409
MLLASSO (PM.3L.GPMs) (*α* = 0.2, *β* = 0.35, *γ* = 0.70)	~70	0.2313	0.4913	0.3985	0.7219

GFs.1L (the first layer of genetic features); PT.1L.GPGs (the first layer of predicted expressions of genetically predictable genes); PT.2L.GPGs (the second layer of predicted expressions of genetically predictable genes); PT.1L+2L.GPGs (the combination of PT.1L.GPGs and PT.2L.GPGs); PM.3L.GPMs (the predicted values of genetically predictable metabolites of the third layer).

### Learning PT.1L.GPGs based on genetic markers supervised by transcriptome

**Figure 1:**
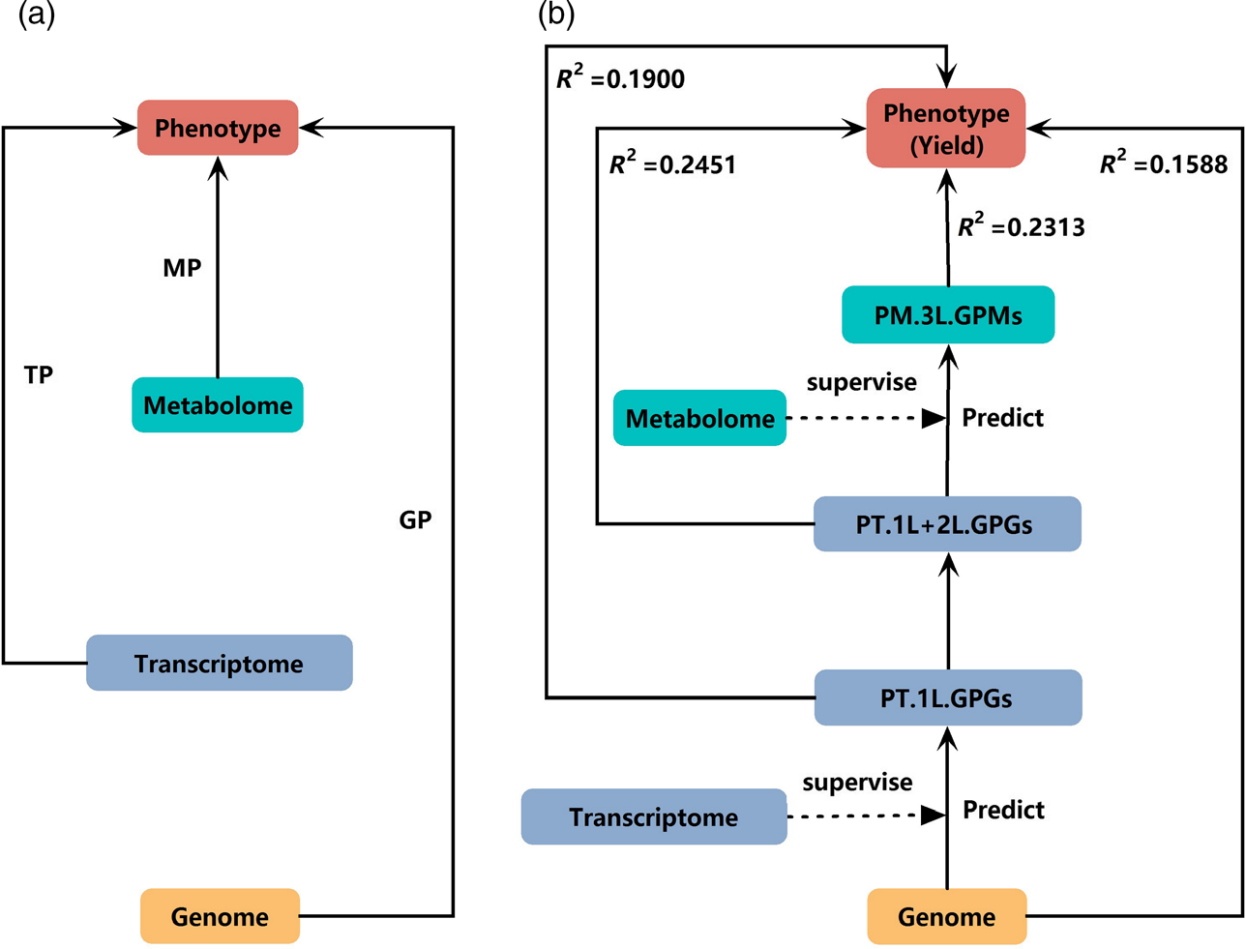
Flow charts of the traditional methods and the new MLLASSO method. (a) Flow chart of the traditional models of genomic prediction (GP), transcriptomic prediction (TP) and metabolomic prediction (MP); (b) flow chart of the new MLLASSO model. (PT.1L.GPGs is the predicted values of ‘genetically predictable genes (GPGs)’ of the first layer; PT.1L+2L.GPGs is the combination of predicted values of GPGs of the first layer and predicted values of GPGs of the second layer; PM.3L.GPMs is the predicted values of ‘genetically predictable metabolites (GPMs)’ of the third layer).

We now focus on an integration analysis model to perform GP. According to the central dogma, genetic information transfers through a hierarchical structure that in turn contains a genomic layer, a transcriptomic layer and a proteomic layer. Recently developed TP or MP model independently used transcriptomic or metabolomic data for prediction, leaving genomic data aside (Figure 1a). To make up the deficiencies, MLLASSO (Figure 1b) integrates genomic, transcriptomic and metabolomic data into a single model that enables us to iteratively learn three layers of GFs by using genetic variants as IV.

We first used a cut‐off criterion of ‘CV > 0.2 (CV, coefficient of variation) and PD > 1 (PD = the 90th percentile―the 10th percentile)’ to screen the whole transcriptome, leaving only 5467 genes passing the criterion and these genes were subject to further analysis (Table S1). Subsequently, we used representative transcriptome consisting of 5467 gene expressions to predict yield with the LASSO method and the predictability increased to 0.5012 (Table 1).

**Figure 2:**
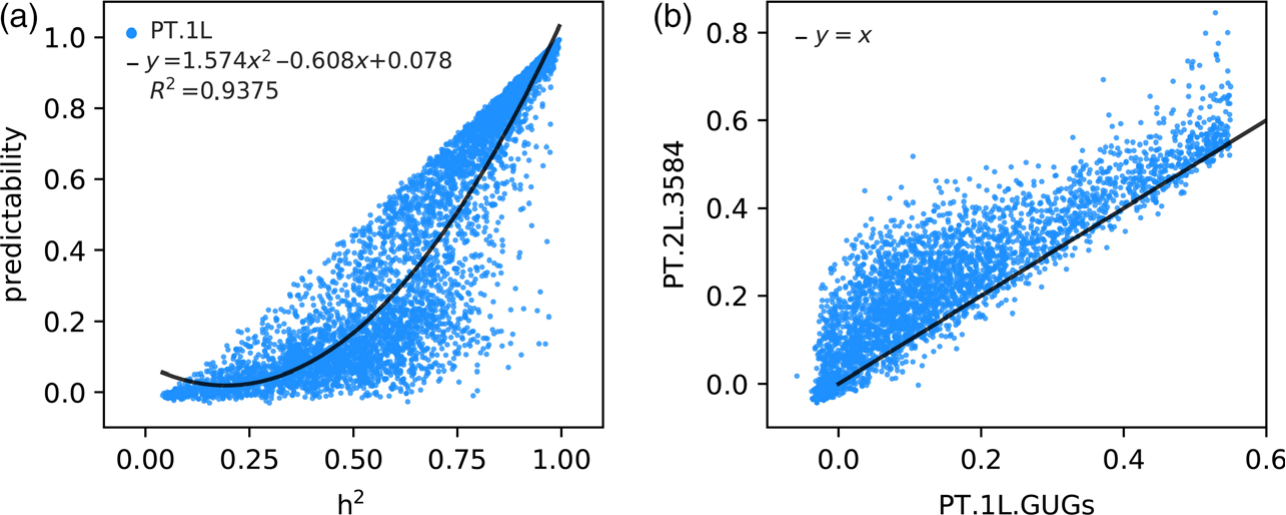
The predicted transcriptome of the first and second layer. (a) The relationship between ‘predictability’ and ‘heritability (*h* ^2^)’ of 5467 genes in PT.1L. (b) Improvement of predictability of PT.1L.GUGs from the first layer to the second layer.

We next try to integrate useful information of representative transcriptome into the GP model. We adopted the IV method (Lin *et al*., 2015) to learn genetic features (GFs.1L) that are determined by genetic markers under the supervision of representative transcriptome. To this end, we used all genomic markers (1619 bins) to predict each transcript of representative transcriptome with the LASSO method and obtained GFs.1L (predicted transcriptome, also denoted by PT.1L). In the whole paper, a 10‐fold CV test was adopted to evaluate the predictability of the trained model. And we here must mention that the 10‐fold CV test was performed in the form of cross‐layer, not in the form of within‐layer. Cross‐layer CV first divided the whole samples into ten groups, nine of which were used to train models layer by layer. The remaining one group of samples never appeared in the training process until they were tested with the trained multilayer models. And this training process was repeated for ten times to ensure that each group of samples was tested for just one time. Take group 1 (the first group in the 10‐fold CV; Tables S1 and S2) as an example, we plotted predictability versus heritability (*h* ^2^) (see Experimental Procedures) for the entire 5467 transcripts and found that the two follow a quadratic functional relationship, *predictability *=* *1.574 (*h* ^2^)^2^ − 0.608 (*h* ^2^) + 0.078, with a goodness of fit of 0.9375 (Figure 2a). This result is consistent with a previous finding using predicted transcriptome to perform TWAS on human GTEx data (Gusev *et al*., 2016), and it also indicates that whether or not the expression of a gene can be accurately predicted by genetic variants largely depends on its heritability.

**Figure 3:**
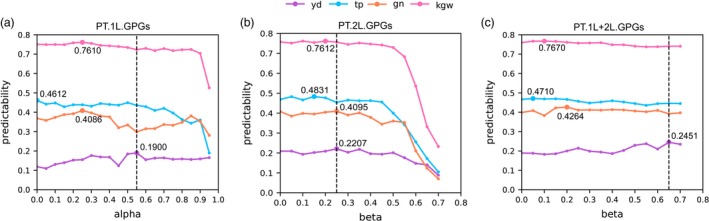
The selection processes of optimal alpha and beta. (a) The variation tendency of predictabilities of four traits while alpha increases from 0.0 to 0.95 in the first layer. (b) The variation tendency of predictabilities of four traits while beta increases from 0.0 to 0.70 of PT.2L.GPGs. (c) The variation tendency of predictabilities of four traits while beta increases from 0.0 to 0.70 of PT.1L+2L.GPGs.

We then asked whether GFs.1L (integrating genomic and transcriptomic information) can improve predictability over GP using only genomic information. Unexpectedly, when we used the whole GFs.1L to predict yield with the LASSO method, the predictability was not improved but was slightly decreased from 0.1588 to 0.1189 via the 10‐fold CV test (Table 1). At first, we were confused with the above result because Lin *et al*. (2015) reported that the predicted transcriptome obtained with genetic variants as IV significantly improved phenotype predictions. Soon afterwards, we noticed that only parts of transcripts were predictable with relatively high predictability (Figure 2a), and we then proposed to use the predicted values of ‘genetically predictable genes (GPGs)’ of the first layer, denoted by PT.1L.GPGs, to predict phenotypes. To this end, we define GPGs as those genes with the condition of their predictabilities > alpha (alpha is a tuning parameter between 0.0 and 0.95) and then we adopted the 10‐fold CV test to determine the optimal value of alpha. Interestingly, the predictability of yield was improved from 0.1588 to 0.1900, representing a 19.65% increasement when using PT.1L.GPGs with *α* = 0.55 (Table 1, Figure 3a). Take group 1 as an example, nearly one‐thirds of the genes (1883/5467~34.44%) were predictable with predictability > 0.55 (Figure 2a, Table S2). The remaining genes (3584/5467~65.56%) with the condition of predictabilities < 0.55 are called ‘genetically unpredictable genes (GUGs)’ of the first layer, denoted by PT.1L.GUGs.

These interesting results offer many implications regarding to phenotype predictions with the predicted transcripts. The observed transcriptome achieves much greater predictability (0.5012) than that (0.1588) of genomic markers, but the predicted transcriptome does not achieve expected predictability to our surprise. The rationale behind this might lie in the great noises of poorly predicted transcripts of PT.1L.GUGs, which harass the feature selection of the LASSO method. However, when the value of alpha is increasing, the corresponding predictability of yield shows a general uptrend and the maximal predictability is achieved as *α* = 0.55 (Figure 3a). When the value of alpha continues to increase, the predictability begins to decrease because the number of PT.1L.GPGs is rapidly decreasing (Figure 3a, Table S2) and not enough number of PT.1L.GPGs would lead to no improvement compared with genomic markers. This means that neither too many nor too few number of PT.1L.GPGs would not achieve improvement of yield predictability, because the former contains many noises of PT.1L.GUGs and the later might discard yield‐related genes. This implies that the key point of accurate yield prediction might depend on the careful selection of yield‐related genes and the ability of capturing information regarding gene interactions. Finally, it is noteworthy that the predictability of 0.1900 was achieved only by genomic markers (genomic markers→PT.1L.GPGs→yield), being >0.1588, the predictability of genomic prediction (genomic markers→yield).

### Learning PT.2L.GPGs based on combinatorial genetic features

We now focus on PT.1L.GUGs that were not predictable with genetic variants in the first layer. In this case, we speculate that their expressions may be affected by other regulator genes (such as PT.1L.GPGs). To confirm our conjecture, we asked whether or not the predictabilities of PT.1L.GUGs can be improved by combining genomic markers and the predicted expressions of PT.1L.GPGs as a new input.

Let us continue to take group 1 as an example, 3113 genes out of 3584 PT.1L.GUGs (3113/3584~86.86%) have increased their predictabilities and the average gain is 0.0764 (Figure 2b, Table S3). As the same with the first layer, we define genetically predictable genes of the second layer (PT.2L.GPGs) as those genes with the condition of their second‐layer predictabilities > beta (beta is a tuning parameter between 0.0 and 0.70) and then we adopted the 10‐fold CV test to determine the optimal value of beta. Importantly, 138 genes out of 3584 PT.1L.GUGs (138/3584~3.85%) (Table S4) have achieved significant improvement of predictability from <0.55 to >0.55, which means that they have changed their status from GUG to GPG. When comparing with the differences in predictability between PT.1L.138 and PT.2L.GPGs (both have the same 138 genes, Table S4), the maximal gain is 0.3208 and the average gain is 0.1026. Another problem that cannot be ignored is that a majority of 3584 PT.1L.GUGs (3446/3584~96.15%), called genetically unpredictable genes of the second layer (PT.2L.GUGs), still fail to be predicted. In summary, the second layer of LASSO allows us to integrate genomic markers and predicted expressions of GPGs into prediction of GUGs expressions and the majority of GUGs show predictability improvements in some degree and the improvements for some GUGs are significant by changing their status from GUGs to GPGs.

We next asked whether or not more GPGs genes would further improve predictability. To this end, we first used PT.2L.GPGs (depending on beta) to predict yield and monitored the predictability while beta increases. As a result, the predictability achieved the maximum of 0.2207 when *β* = 0.25 (Table 1, Figure 3b), where PT.2L.GPGs have 1776 genes. Importantly, we then combined PT.1L.GPGs (fixing *α* = 0.55) and PT.2L.GPGs (depending on beta) to build a second layer of genetic feature of PT.1L+2L.GPGs. When we used PT.1L+2L.GPGs to predict yield, the predictability showed a general uptrend as beta increasing from 0.0 to 0.70 and achieved the maximum of 0.2451 when *β* = 0.65 (Table 1, Figure 3c). This time, a further increase of 29.00% and a total increase of 54.35% have been achieved, suggesting that PT.1L+2L.GPGs are more powerful in predicting yield of rice.

### Learning PM.3L.GPMs based on PT.1L+2L.GPGs supervised by metabolome

Having provided a variety of evidences to support the conclusion that multiple layers of learned genetic features can improve GP for yield, we now further examine whether or not the conclusion applies to other traits. We constructed the third layer of LASSO to learn the third layer of genetic features GFs.3L based on PT.1L+2L.GPGs (depending on the values of both alpha and beta) supervised by the observed metabolome (1000 metabolomic traits, where the expression level of each metabolite was treated as a quantitative trait) (Table S5). At the same time, predictions of metabolomic expressions from genomic markers were simultaneously carried out for comparisons.

**Figure 4:**
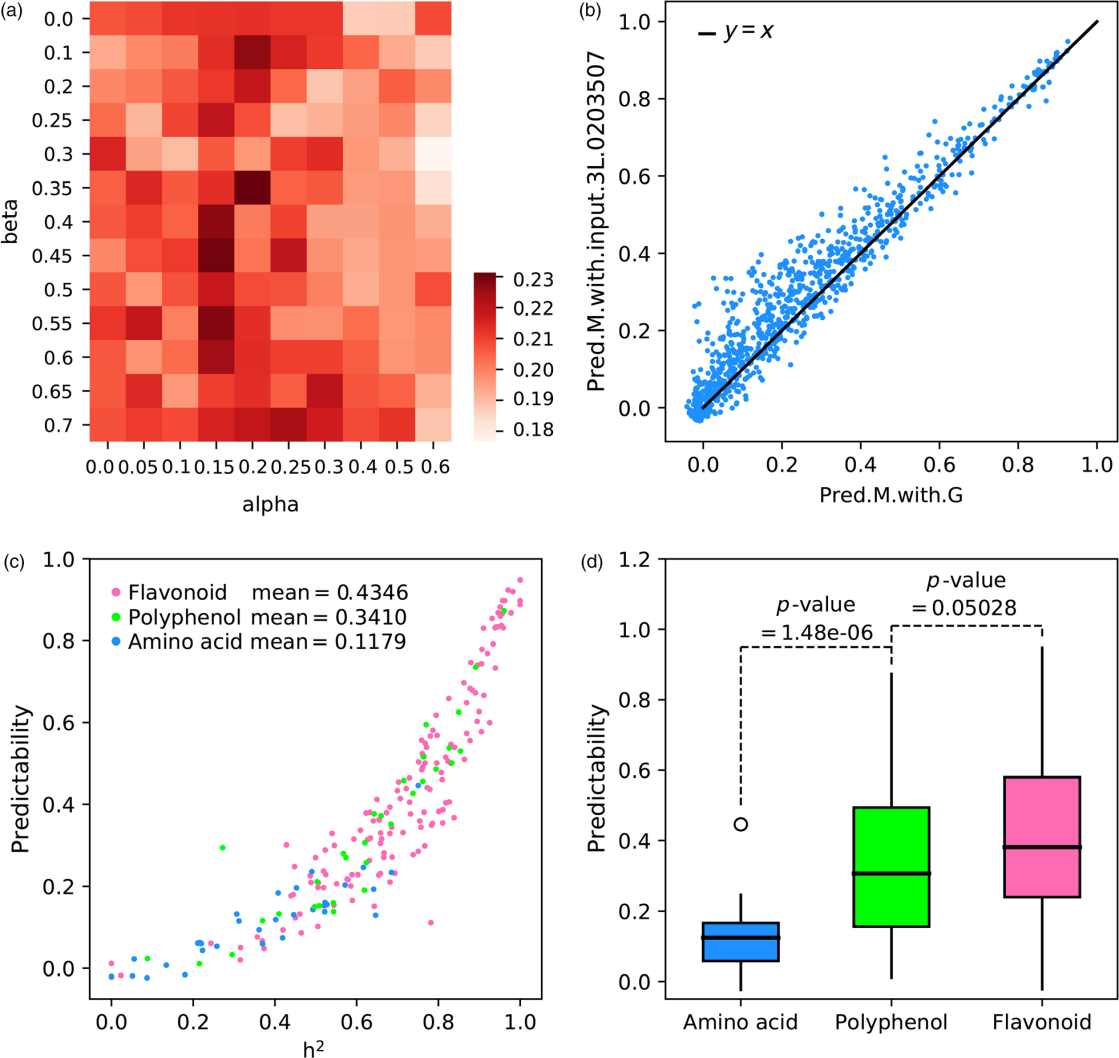
The prediction results of metabolome (1000 metabolites) using genome and PT.1L+2L.GPGs. (a) The heatmap of optimal search on combination of alpha and beta; (b) the promotion of predictabilities of 1000 metabolites from using genomic markers (Pred.M.with.G) to using input.3L of *α* = 0.2, *β* = 0.35 and *γ* = 0.7 (Pred.M.with.input.3L.0203507); (c). predictabilities of the largest three metabolite classes: flavonoid, ployphenol and amino acid; (d). box plots of predictabilities of three metabolite classes and pairwise statistical tests.

Unexpectedly, when we used PT.1L+2L.GPGs with the optimal values of *α* = 0.55 and *β* = 0.25 of the second layer to predict 1000 metabolites and then used their predicted values to predict yield trait, the predictability of yield (0.1898) was not significant improved compared with the predictability of the second layer. We noted that the values of *α* = 0.55 and *β* = 0.25 were optimal for accurate prediction of gene expressions and were not necessarily optimal for accurate prediction of metabolites. Meanwhile, we define genetically predictable metabolites of the third layer (PM.3L.GPMs) as those metabolites with the condition of their third‐layer predictabilities > gamma (gamma is a third tuning parameter between 0.0 and 0.90) and then we adopted the 10‐fold CV test to determine the optimal value of gamma. For this reason, we performed a grid search on the combination of alpha, beta and gamma to determine the optimal combination of alpha, beta and gamma for metabolite prediction (Figure 4a). As a result, a maximal predictability of 0.2313 was achieved on the combination of *α* = 0.2, *β* = 0.35 and *γ* = 0.70. Although the predictability of 0.2313 did not further improve yield prediction, we still found some interesting results. On the one hand, the finding of *γ* = 0.70 means that we need to select genetically predictable metabolites to predict yield. And when we select PT.1L.GPGs and PT.2L.GPGs to make metabolite prediction, the finding of *α* = 0.2 and *β* = 0.35 implies a trade‐off between the predictabilities and the number of selected genes. On the other hand, when comparing predictabilities of metabolites by using genome, PM.3L.GPMs showed some superiorities by achieving that the majority of 1000 metabolites (703/1000 = 70.3%) were more predictable than using genome alone (Figure 4b, Table S5). The maximal improvement of predictability was 0.3093, and the average promotion was 0.034.

According to a previous study, 415 out of 1000 metabolites were annotated into 18 different metabolite classes (Gong *et al*., 2013). We next ask whether different metabolite classes would have distinctive predictabilities. To this end, we chose three largest metabolite classes: flavonoid (150 metabolites), polyphenol (31 metabolites) and amino acid (32 metabolites); their corresponding predictabilities are shown in Figure 4c with different colours. The mean values of predictabilities within the three classes are 0.4346, 0.3410 and 0.1179, respectively, and pairwise statistical tests (*t*‐test) show that the differences are statistically significant (Figure 4d), suggesting that metabolites of different classes have different predictive properties, and the secondary metabolites, like flavonoid, are well predicted whereas amino acid metabolites are not.

Moreover, the prediction results of Tiller and Grain are consistent with those of yield—predictabilities of all three agronomic traits (except KGW) have been improved with PT.1L+2L.GPGs or PM.3L.GPMs over GP (Table 1). More precisely, the improvement of Grain has a pattern similar to that of yield—genome alone has the lowest predictability, the observed transcriptome has the highest predictability, and either PT.1L+2L.GPGs or PM.3L.GPMs have intermediate predictability. In contrast, GPs for Tiller and KGW are more efficient than TPs, but prediction using PT.1L+2L.GPGs or PM.3L.GPMs is more efficient than both for Tiller. In summary, PT.1L+2L.GPGs or PM.3L.GPMs are two predictive feature sets of various types of phenotypes, including phenotypes of agronomic traits and phenotypes of metabolomic traits.

We comprehensively analysed the whole results and concluded that, for polygenic traits controlled by hundreds or thousands of genes with small effects, for example yield, GP is unsatisfactory and TP or MP is much more efficient, and these polygenic traits are good candidates for TP or MP. When transcriptome and metabolome are unavailable, MLLASSO using PT.1L+2L.GPGs or PM.3L.GPMs is a beneficial complement to GP. On the other hand, for traits affected by a few genes with large effects, for example KGW, GP is often the best choice. The conclusion is that genomic prediction facilitated by MLLASSO might be more efficient than the single layer of genomic prediction. More importantly, PM.3L.GPMs (predicted metabolome) are computed as the linear combination of PT.1L+2L.GPGs (predicted gene expression), which contains useful information of gene interactions, suggesting that MLLASSO has learned higher order information of gene interactions.

### Predictabilities of gene expression are associated with the regulation roles of genes

We further try to understand why different genes show different predictabilities. We focused on two gene sets, PT.1L.GPGs and PT.2L.GPGs (Table S6), and conducted comprehensive comparisons between them based on a previously reported eQTL analysis from the same population (Wang *et al*., 2014). Of the 13 647 eQTLs reported, 5079 are *cis*‐eQTLs (37.2%) and 8568 are *trans*‐eQTLs (62.8%). Taking group 1 as an example, a detailed analysis of PT.1L.GPGs showed that, out of 1883 PT.2L.GPGs, 1317 are *cis*‐eQTLs and 515 are *trans*‐eQTLs, that is more than seventy per cent (1317/(1317 + 515) ~ 71.89%) of PT.1L.GPGs are *cis*‐eQTLs (Table 2). On the contrary, out of 1767 PT.2L.GPGs, 644 genes are *cis*‐eQTLs and 957 genes are *trans*‐eQTLs, that is nearly sixty per cent (957/(957 + 644) ~ 59.78%) of PT.2L.GPGs are *trans*‐eQTLs (Table 2).

**Table 2:** Statistics for genetically predictable genes (GPGs) of two layers on their eQTL results

PT.1L.GPGs (1883 genes)				PT.2L.GPGs (1767 genes)			
Cis\Trans	Non‐Trans	Trans	Total	Cis\Trans	Non‐Trans	Trans	Total
Non‐Cis	367	201		Non‐Cis	438	685	
Cis	1001	314	1317	Cis	372	272	644
Total		515		Total		957	

PT.1L.GPGs (the first layer of predicted expressions of genetically predictable genes); PT.2L.GPGs (the second layer of predicted expressions of genetically predictable genes).

In summary, an eQTL analysis demonstrated that PT.1L.GPGs are mostly *cis*‐eQTL genes and PT.2L.GPGs are mostly *trans*‐eQTL genes. This together with the definition of *cis*‐eQTL genes (whose expressions are determined by neighbouring genetic variants) give a rational explanation about why the expressions of PT.1L.GPGs are well predicted with genomic markers. In contrast, the variation of *trans*‐eQTL gene within the population is not only determined by the cis‐regulatory elements of itself but also affected by other genes, which explains why the expressions of PT.2L.GPGs are poorly predicted with genomic markers only.

### Transcriptional factor families are differentially enriched in GPGs

**Figure 5:**
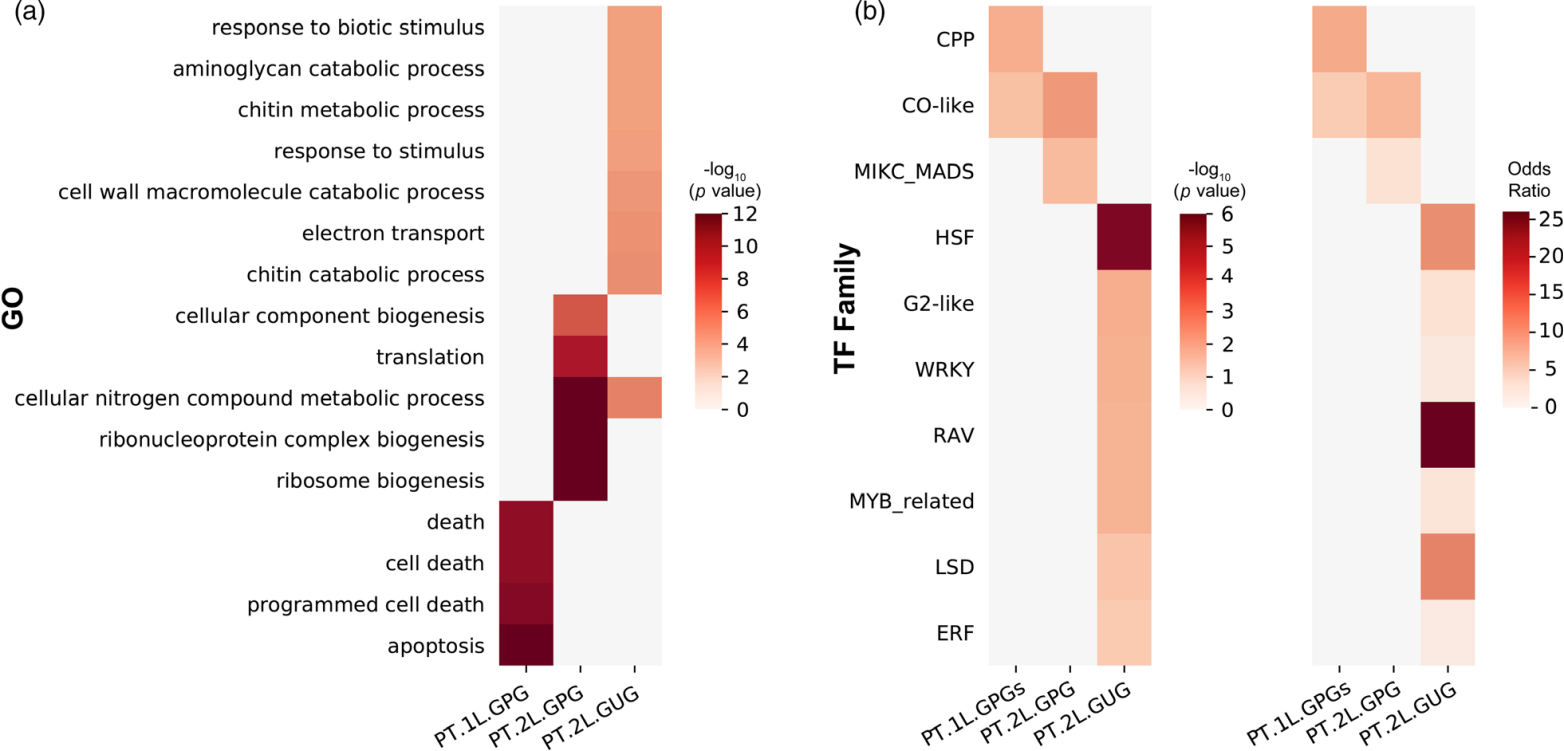
Enriched GO terms and enriched TF families of PT.1L.GPGs, PT.2L.GPGs and PT.2L.GUG. (a) Enriched GO terms of PT.1L.GPGs, PT.2L.GPGs and PT.2L.GUG; (b). Enriched TF families of PT.1L.GPGs, PT.2L.GPGs and PT.2L.GUG.

To provide a biological interpretation, two well‐predicted gene sets, that is PT.1L.GPGs and PT.2L.GPGs (Table S6), and a poorly predicted gene set PT.2L.GUG were used to perform GO enrichment analyses with a comprehensive bioinformatics tool ‘agriGO’ (Tian *et al*., 2017). As a result, four nonredundant GO terms were significantly enriched against the background in PT.1L.GPGs (Figure 5a, Table S7). Notably, they are mainly fundamental biological processes including cell death and apoptosis. In contrast, when considering PT.2L.GPGs, overall 8 GO terms were significantly enriched (Table S7). The top five significant GO terms including ribosome biogenesis, cellular nitrogen compound metabolic process, ribonucleoprotein complex biogenesis, translation and cellular component biogenesis were chosen to show representative results (Figure 5a). Interestingly, this time more downstream biological processes are enriched and some of them are associated with metabolic process, which may be an explanation why the majority of metabolites are better predictable using PT.1L+2L.GPGs when compared with the cases using genome.

We next performed TF family enrichment analyses on three gene sets, that is PT.1L.GPGs, PT.2L.GPGs and PT.2L.GUGs by using a comprehensive resource of plant TF database ‘PlantTFDB 4.0’ (Jin *et al*., 2017). First, a direct statistic shows that the numbers of TF in the three gene sets are 50 (out of 1883), 73 (out of 1767) and 106 (out of 1817) respectively, and the corresponding TF percentages are 2.655%, 4.131% and 5.834%, respectively. This implies that it is relatively hard to accurately predict TF expressions as a whole (when compared with 4.617% of TF percentage of background, i.e. the whole gene set).

More importantly, we want to know among all the 56 TF families which families are enriched in each gene set. To this end, we performed a TF family enrichment analysis with the Fisher's exact test on each gene set. We listed all the statistical results in Table S8, from which we found that a TF family termed ‘CO‐like’ was significantly enriched in both PT.1L.GPGs and PT.2L.GPGs with *P*‐value < 0.01 and odds ratio > 1 (Figure 5b, Table S8). As an evidence, Griffiths *et al*. (2003) reported that CO (CONSTANS) gene generally contains a famous CCT (CO, CO‐like, TOC1) domain and acts between the circadian clock and genes controlling meristem identity. The CCT proteins were also reported to be key factors in regulatory networks controlling photoperiodic regulation of flowering (Gangappa and Botto, 2014). Notably, a famous gene named ‘*Ghd7*’ who is a major regulator affecting a group of yield‐related traits including number of grains per panicle, plant height and heading date was also reported to encode a CCT domain protein (Xue *et al*., 2008). Another two enriched TF families of PT.1L.GPGs are ‘MIKC_MADS’ (a gene family controlling flower development in plant) (Nam *et al*., 2003) and ‘CPP’ (a gene family playing an important role in the development of reproductive tissue and control of cell division) (Yang *et al*., 2008), both of which are yield‐related TF families (Figure 5b). In contrast, the enriched TF families of PT.2L.GUGs are mainly gene families responding to environmental stimuli (Figure 5b, Table S8). For example, ‘ERF’ and ‘RAV’ families were reported as ethylene response factor that are involved in many diverse functions in cellular processes, such as hormonal signal transduction and response to biotic and abiotic stresses (Fukao *et al*., 2006; Xu *et al*., 2006); ‘HSF’ family genes are the major regulators of the plant heat stress response (Koskull‐Döring *et al*., 2007).

In summary, TF enrichment analyses demonstrated that TF expressions are less predictable as a whole, but some yield‐related TF families, such as ‘CO‐like’, ‘MIKC_MADS’ and ‘CPP’, were still enriched in GPGs. On the contrary, gene families responding to environmental stimuli, such as ‘ERF’ and ‘HSF’, were enriched in GUGs. This may explain why the predictability of yield using PT.1L.GPGs or PT.2L.GPGs is much better than that using genome alone and why expressions of GUGs are hard to predict.

## Discussion

Integration analysis based on multiple omic data is a promising way to enable us systematically identify hidden mechanisms in which multiple intermediate variables are involved in complex traits. In this paper, we proposed an innovative approach, that is MLLASSO, that enables us to learn three layers of genetic features, PT.1L.GPGs, PT.1L+2L.GPGs and PM.3L.GPMs, supervised by observed transcriptome and metabolome. In comparisons with classic genomic prediction approaches, MLLASSO has two main advantages:

### Directed learning with guidance of intermediate omic data

The classic LASSO method adopts a simple learning strategy by selecting most important markers and then taking their linear combination as a predicted value of a concerning trait. In its algorithm implementation, the LASSO method tries to find an optimal marker combination by selecting markers highly correlated with a given trait. Unfortunately, it may have a high risk of selecting false markers who are neighbouring to the determinant marker due to linkage disequilibrium (LD). For example, a QTL named *Ghd7* located in Bin1006 was reported as a major gene for serval yield‐related traits (Xue *et al*., 2008), but the LASSO method selects a total of 52 bins in which Bin1004, not Bin1006, is the top one with the highest correlation (Table S9). The reason why the LASSO method selects Bin1004 instead of Bin1006 is due to its higher correlation with the yield (0.3028) than that of Bin1006 (0.2912). Between genome and phenotype, there is a huge gap in which multiple layers of intermediate omics including transcriptome and metabolome exist. Without guidance of these intermediate omic data, a machine does not have a specific direction to identify the determinant markers among a group of highly correlated markers. It may take a simple strategy of selecting the most correlated ones and dropping others. We summarized this kind of learning strategy as ‘undirected learning’.

MLLASSO integrates multiple layers of omic data into a single model that enable us to learn three layers of intermediate variables or genetic features. In each layer, each genetic feature is learned under the supervision of one specific gene expression or metabolite. This gives the machine a specific direction to optimize linear combinations of genetic markers and then to form a module of biological network. When predicting a phenotype using these learned modules, recomposition between them might learn high order information of gene interactions. This is what we called a ‘directed learning’ strategy and it is the most significant contribution of the current study.

### Robust prediction with trait‐related bins mapped by backtracking

MLLASSO enables us to map real trait‐related bins with backtracking. MLLASSO runs a forward prediction manner in turn passing genome, transcriptome, metabolome and phenotype. In contrast, a backtracking technology allows us to select a path of each genetic bin layer by layer in the opposite direction. Using this approach, we backtracked all the selected bins involved in our MLLASSO prediction model. Furthermore, we computed the product of all coefficients recorded in each backtracking path of each selected bin and finally summed up all the product values belonging to the same selected bin. Let us take the yield trait as an example, a backtracking approach identified a total of 447 genetic bins and calculated their coefficients (Table S9). Interestingly, Bin1006 that contains *Ghd7* gene was ranked the first with the greatest coefficient (1.2883) in the backtracking result. Notably, the second bin was Bin893 with a coefficient of 0.7162, which contains another reported gene *hd1* controlling response to photoperiod (Yano *et al*., 2000). In comparison with the selected bins by the single layer LASSO, neither Bin1006 nor Bin893 was selected; instead, Bin1004 and Bin890 were selected. One can see that a backtracking approach of MLLASSO is more robust in selecting real trait‐related genetic markers compared with the classic LASSO method.

Finally, the proposed MLLASSO approach provides a practical strategy to deal with some common problems: heterogeneity between different data source, imputation of missing values (using learned intermediate variables), and sparse selection of significant variables and their biological interpretations. We believe that it will be of general interests to a broad field of integrative analysis studies. The current study contributes to the field of genomic selection with an innovative prediction framework of directed learning as well as several specific technologies including backtracking. We believe that application of the MLLASSO model to various species will accelerate mechanism discoveries of complex traits and molecular breeding of plants.

## Experimental procedures

### Data sets

The data set consists of 210 RILs of rice that were derived from nine rounds of selfings from the cross between Zhenshan 97 and Minghui 63, two elite *indica* rice varieties (Hua *et al*., 2003). The 210 RILs were sequenced using next‐generation sequencing technology (Xie *et al*., 2010), and the sequencing results were confirmed with traditional RFLP/SSR markers (Yu *et al*., 2011). As a result, a total of 270 820 high‐quality SNPs were identified as the genotypes of each individual. The SNPs were subsequently combined into 1619 bins. Within a bin, all markers segregate with the same pattern. The genomic data are represented by a 210 × 1619 feature matrix, where each element (*x*_*ij*_) is a numerically coded genotype for individual *i* at bin *j*.

Gene expressions of transcriptomic data were measured by extracting RNA collected from flag leaves in the microarray sequencing platform. A total of 24 994 genes were recorded from all the 210 RILs, forming a 210 × 24 994 feature matrix (Wang *et al*., 2014). The metabolomic data consist of 683 metabolites measured from flag leaves and 317 metabolites measured from germinated seeds. The 683 + 317 = 1000 metabolites form a 210 × 1000 matrix for metabolomic traits (1000 response variables) (Gong *et al*., 2013). For these intermediate phenotypes, the heritability of each gene expression or metabolite was calculated as the broad‐sense heritability. Four important agronomic traits were collected from the same population, including yield per plant (Yield), tiller number per plant (Tiller), grain number per panicle (Grain) and 1000 grain weight (KGW) (Data set S1). The traits were obtained from a field experiment on the Farm of Huazhong Agricultural University, Wuhan, China (Yu *et al*., 2011).

### The LASSO method

The LASSO method was first invented by Tibshirani (1996) for a feature selection task that employs regularization techniques to perform variable selection and prediction, which makes it a widely used statistical learning method in the past decade. The LASSO method often yields a *sparse* model that involve only a small subset of variables or features, which makes it more interpretable than other methods (such as GBLUP or ridge regression) (Tibshirani, 1996). In this study, the LASSO method was implemented with the GLMNET/R package (Friedman *et al*., 2010).

### Cross‐validation and predictability

The tuning parameter *λ* in the LASSO method is determined via a 10‐fold CV method to minimize the predicted residual sum of squares (PRESS). The predictability is defined as follows:

(1) $$R^{2}=1-\frac{\mathrm{PRESS}}{\mathrm{SS}},$$

where $SS=\sum_{i=1}^{n}{\left(y_{i}-\overset{¯}{y}\right)}^{2}$ is the total sum of squares and $PRESS=\sum_{i=1}^{n}{\left(y_{i}-{\hat{y}}_{i}\right)}^{2}$ where ${\hat{y}}_{i}$ is the predicted trait value using training samples excluding the group containing *y*_*i*_.

### MLLASSO

The main idea of MLLASSO was inspired by the instrumental variable (IV) analysis from a published work (Lin *et al*., 2015) where genomic markers were treated as the instrumental variables and gene expressions as the intermediate variables close to the phenotypes (trait values) of interest. The method was implemented in a two‐stage regularization (2SR) process where the first stage is represented by prediction of gene expressions using the instrumental variables and the second stage is represented by prediction of trait values using the predicted expressions of genes. The 2SR model is described as follows (Lin *et al*., 2015).

Let *Y*_*n *× 1_ be a vector of phenotypes, *X*_*n *× *p*_ = (*X*_1_,..,*X*_p_) be a feature matrix of gene expressions and *Z*_*n *× *p*_ = (*Z*_1_,…,*Z*_q_) be a feature matrix of genomic markers, where *n* is the sample size, *p* is the number of genes and *q* is the number of genomic markers. Using genotypes as instrumental variables, the linear model jointly fits the data (*Y*,* X*,* Z*) is described below,

(2) $$\left\{\begin{array}{c} Y=X\beta_{0}+\eta \\ X=Z\Gamma_{0}+E \end{array}\right.,$$

where *β*_0_ and Γ_0_ are regression coefficients with dimensions *p* × 1 and *q* × *p,* respectively, *η* and *E* are random errors with dimensions *n* × 1 and *n* × *p*, respectively. Lin *et al*. (2015) used a two‐stage least squares (2SLS) method to choose an optimal sparse subset of *β*_*0*_ and Γ_0_. Through strict mathematical derivation and a large scale of simulation test, they claimed that the 2SR method was reliable and powerful for genomic prediction.

The framework of our MLLASSO is similar to the 2SR method, but we created two layers of predicted transcriptome and one more layer of predicted metabolome whereas 2SR only has one layer. Let *Z*_*n *× *q*_ = (*Z*_1_,..,*Z*_*q*_) be the feature matrix of genome and *X*_*n *× *q*_ = (*X*_1_,..,*X*_*p*_) be the feature matrix of transcriptome. The first layer LASSO uses a regularization methodology with *L*_1_‐penalty to predict *X* based on *Z*. Let 0 < *α*<1 be a discrimination threshold, based on which we define ${\hat{X}}^{\mathrm{GPG}.1L}=\left({\hat{X}}_{1}^{\mathrm{GPG}.1L},\cdots ,{\hat{X}}_{k}^{\mathrm{GPG}.1L}\right)$ as a feature matrix of predicted expressions of GPG.1L with predictability > *α* in the first layer, where *k* is the number of GPG.1L. We then define $X_{n\times m}^{\mathrm{GUG}.1L}=\left(X_{1}^{\mathrm{GUG}.1L},\cdots ,X_{m}^{\mathrm{GUG}.1L}\right)$ as a feature matrix of observed expressions of GUG.1L with probability ≤ *α* in the first layer LASSO, where *m* is the number of GUG and *k + m = p*.

The second layer of LASSO uses a regularization methodology with *L*_1_‐penalty to predict *X* ^GUG^ based on the combination of *Z* and ${\hat{X}}^{\mathrm{GPG}.1L}$. Let 0 < *β*<1 be another discrimination threshold, based on which we define ${\hat{X}}^{\mathrm{GPG}.2L}=\left({\hat{X}}_{1}^{\mathrm{GPG}.2L},\cdots ,{\hat{X}}_{l}^{\mathrm{GPG}.2L}\right)$ as a feature matrix of predicted expressions of GPG.2L with predictability > *β* in the second layer, where *l* is the number of GPG.2L.

Let *M*_*n *× *t*_ = (*M*_1_,..,*M*_*t*_) be the feature matrix of metabolome, where *t* is the number of metabolites. The third layer of LASSO uses a regularization methodology with *L*_1_‐penalty to predict *M* based on the combination of ${\hat{X}}^{\mathrm{GPG}.1L}$ and ${\hat{X}}^{\mathrm{GPG}.2L}$. MLLASSO jointly models the data (*Y*,* M*,* X* ^GUG.1L^, *X*,* Z*) using

(3) $$\left\{\begin{array}{c} Y=\hat{M}\delta_{0}+\chi \\ M={\hat{X}}^{\mathrm{GPG}.1L}\beta_{0}+{\hat{X}}^{\mathrm{GPG}.2L}\Psi_{0}+\eta \\ X^{\mathrm{GUG}.1L}={\hat{X}}^{\mathrm{GPG}.1L}\Omega_{0}+Z\Phi_{0}+\varepsilon \\ X=Z\Gamma_{0}+E \end{array}\right.$$

where *Y*_*n *× *1*_ is a vector of phenotypes, *δ*_0_, *β*_0_, Ψ_0_, Ω_0_, Φ_0_ and Γ_0_ are all regression coefficients with dimensions *t × *1, *k* × *t*,* l* × *t, k* × *m, q* × *m* and *q* × *p,* respectively, and *χ*,* η*,* ε* and *E* are all random errors with dimensions *n *×* *1, *n* × *t*,* n* × *m* and *n* × *p,* respectively. The regularization method with *L*_1_‐penalty applies to all three layers and each layer selects an optimal set of sparse representatives of the regression coefficients. In this particular study, *n *=* *210, *q *=* *1619, *p *=* *5467, *t *=* *1000. And when *α *= 0.55, *β *= 0.25, we have *k *=* *1883, *m *=* *3584 and *l *=* *1767.

### Gene and TF family enrichment analysis

We used two bioinformatics tools to perform gene ontology analysis and TF family enrichment analysis. AgriGO (Tian *et al*., 2017) is a web‐based gene ontology analysis tool (http://bioinfo.cau.edu.cn/agriGO/analysis.php) that was specifically designed for agricultural species. It currently supports 45 species including Arabidopsis thaliana, Oryza sativa and Zea mays, and their overall 292 data types. PlantTFDB 4.0 used gene annotation information from MSU(v7) database (Kawahara *et al*., 2013) to identify 2408 TFs of Oryza sativa subsp.Japonica. Family assignment rules further classified them into 56 families, whose list can be downloaded at the following website http://planttfdb.cbi.pku.edu.cn/index.php?sp=Osj.

### Backtracking

From the four simultaneous Equations in (3), we performed backtracking using

(4) $$\begin{array}{cc} Y & =\hat{M}\delta_{0}+\chi \\ & =\delta_{0}\left({\hat{X}}^{\mathrm{GPG}.1L}\beta_{0}+{\hat{X}}^{\mathrm{GPG}.2L}\Psi_{0}+\eta \right)+\chi \\ & =\delta_{0}\left(\left({\hat{X}}^{\mathrm{GPG}.1L}\Omega_{0}+Z\Phi_{0}+\varepsilon \right)\Psi_{0}+{\hat{X}}^{\mathrm{GPG}.1L}\beta_{0}+\eta \right)+\chi \\ & =\delta_{0}\left(\left(\Omega_{0}+\Psi_{0}\right){\hat{X}}^{\mathrm{GPG}.1L}+Z\Phi_{0}\Psi_{0}+\varepsilon \Psi_{0}+\eta \right)+\chi \\ & =\delta_{0}\left(\left(\Omega_{0}+\Psi_{0}\right)\left(Z\Gamma_{0}^{\mathrm{GPG}.1L}+E\right)+Z\Phi_{0}\Psi_{0}+\varepsilon \Psi_{0}+\eta \right)+\chi \\ & =\left[\delta_{0}\left(\Omega_{0}+\Psi_{0}\right)\Gamma_{0}^{\mathrm{GPG}}+\delta_{0}\Phi_{0}\Psi_{0}\right]Z+\left[\delta_{0}\left(\Omega_{0}+\Psi_{0}\right)E+\delta_{0}\varepsilon \Psi_{0}+\delta_{0}\eta +\chi \right]\\ & =\Delta Z+e \end{array}$$

which deduces the direct linear relationship between phenotype *Y* and genotype *Z*.

## Conflict of interest

The authors declare no conflict of interest.

## Supporting information


**Table S1** Heritabilities and predictabilities of 5467 gene expressions in first layer.
**Table S2** Predictabilities of ten groups of 10‐fold‐cross‐validation.
**Table S3** Comparisons of predictabilities of 3584 PT.1L.GUG in the first layer and in the second layer.
**Table S4** Comparisons of predictabilities of 138 PT.2L.GPG in the first layer and in the second layer.
**Table S5** Comparisons of predictabilities of 1000 metabolites based on genome and PT.1L+2L.GPGs.
**Table S6** Gene IDs of PT.1L.GPGs. and PT.2L.GPGs.
**Table S7** Results of GO enrichment analyses of PT.1L.GPG, PT.2L.GPG and PT.2L.GUG.
**Table S8** TF family enrichment analyses of PT.1L.GPG, PT.2L.GPG and PT.2L.GUG. TF family names along with their pfam ID and enrichment results of *P*‐value and odd.ratio are shown.
**Table S9** The list of selected bins and their coefficients of backtracking of MLLASSO. The list of selected bins and their coefficients of single layer LASSO are also showed as a comparison.

**Data set S1** Four agronomic traits of 210 RILs rice population.
